# Supplementary material for: The interactome of intact mitochondria by cross-linking mass spectrometry provides evidence for coexisting respiratory supercomplexes
Source: Mol Cell Proteomics. 2017 Dec 8;17(2):216–32. doi: 10.1074/mcp.RA117.000470 (PMC5795388; doi:10.1074/mcp.RA117.000470)
Supplement: Supplemental Data [file supp_RA117.000470_133922_0_supp_23978_fzffwf.zip › spectra_annotation/mito_DR_spectra_annotation/244-1-2-1-5-1.pdf]

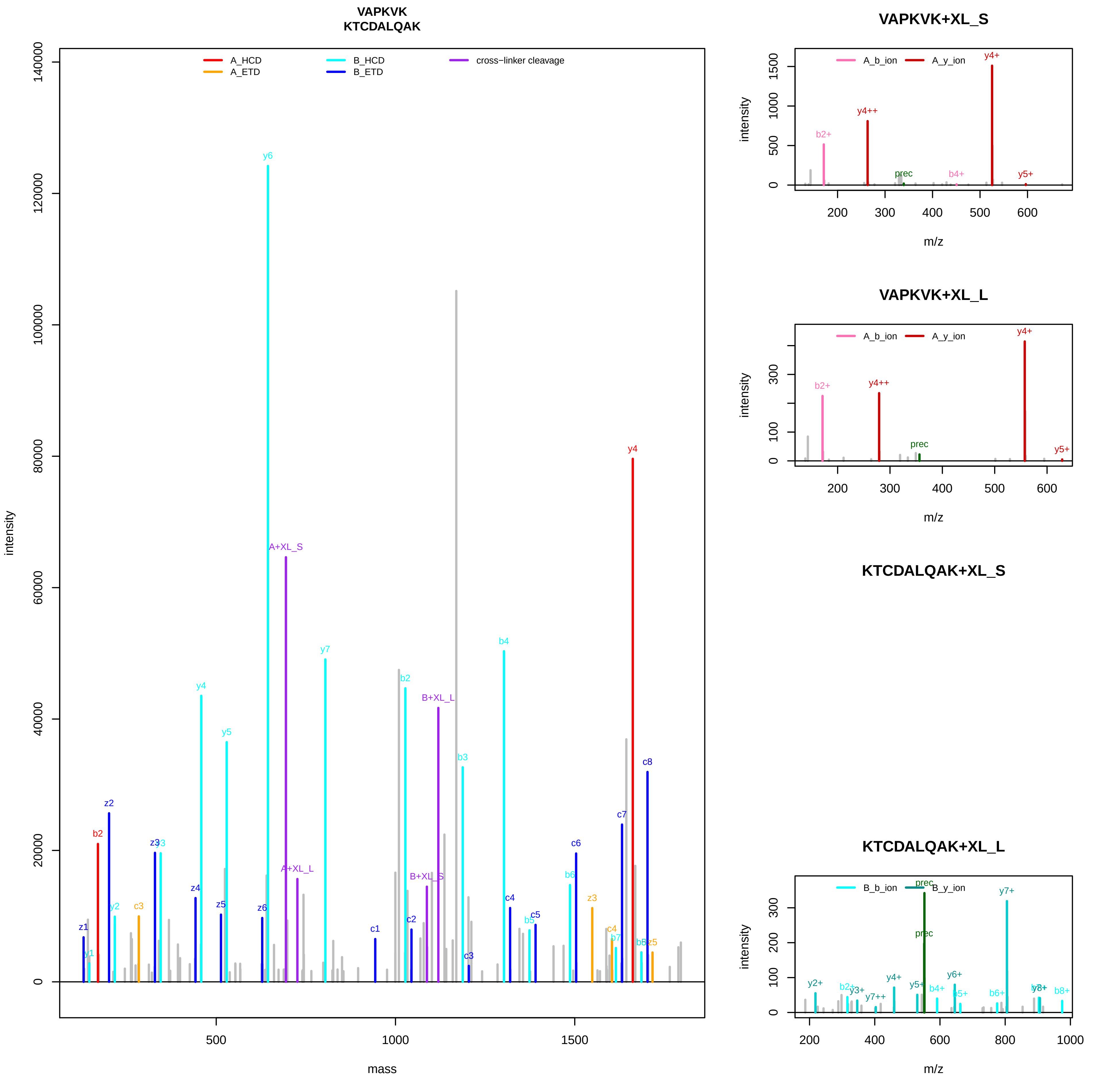

VAPKVK
KTCDALQAK
A_HCD
A_ETD
B_HCD
B_ETD
cross−linker cleavage
intensity
mass
0
20000
40000
60000
80000
100000
120000
140000
500
1000
1500
z1
y1
b2
z2
y2
c3
z3
y3
z4
y4
z5
y5
z6
y6
A+XL_S
A+XL_L
y7
c1
b2
c2
B+XL_S
B+XL_L
b3
c3
b4
c4
b5
c5
b6
c6
z3
c4
b7
c7
b5
c8
y4
z5
VAPKVK+XL_S
A_b_ion
A_y_ion
b2+
y4++
prec
b4+
y4+
y5+
0
500
1000
1500
200
300
400
500
600
m/z
VAPKVK+XL_L
A_b_ion
A_y_ion
b2+
y4++
prec
y4+
y5+
0
100
300
200
300
400
500
600
m/z
KTCDALQAK+XL_S
KTCDALQAK+XL_L
B_b_ion
B_y_ion
prec
prec
y7+
y2+
b2+
y3+
y7++
y4+
y5+
b4+
y6+
b5+
b6+
y8+
b8+
0
100
200
300
200
400
600
800
1000
m/z
